# MCM2-7 in Clear Cell Renal Cell Carcinoma: MCM7 Promotes Tumor Cell Proliferation

**DOI:** 10.3389/fonc.2021.782755

**Published:** 2021-12-21

**Authors:** Junneng Zhang, Huanzong Zhang, Yinghui Wang, Qingshui Wang

**Affiliations:** ^1^ Laboratory Medicine Department, The Fifth Hospital of Xiamen, Xiamen, China; ^2^ Key Laboratory of Optoelectronic Science and Technology for Medicine of Ministry of Education, College of Life Sciences, Fujian Normal University, Fuzhou, China

**Keywords:** ccRCC, MCM2-7, WGCNA, Lasso, cell cycle, proliferation

## Abstract

**Background:**

Clear cell renal cell carcinoma (ccRCC) accounts for 60-70% of renal cell carcinoma (RCC) cases. Finding more therapeutic targets for advanced ccRCC is an urgent mission. The minichromosome maintenance proteins 2-7 (MCM2-7) protein forms a stable heterohexamer and plays an important role in DNA replication in eukaryotic cells. In the study, we provide a comprehensive study of MCM2-7 genes expression and their potential roles in ccRCC.

**Methods:**

The expression and prognosis of the MCM2-7 genes in ccRCC were analyzed using data from TCGA, GEO and ArrayExpress. MCM2-7 related genes were identified by weighted co-expression network analysis (WGCNA) and Metascape. CancerSEA and GSEA were used to analyze the function of MCM2–7 genes in ccRCC. The gene effect scores (CERES) of MCM2-7, which reflects carcinogenic or tumor suppressor, were obtained from DepMap. We used clinical and expression data of MCM2-7 from the TCGA dataset and the LASSO Cox regression analysis to develop a risk score to predict survival of patients with ccRCC. The correlations between risk score and other clinical indicators such as gender, age and stage were also analyzed. Further validation of this risk score was engaged in another cohort, E-MTAB-1980 from the ArrayExpress dataset.

**Results:**

The mRNA and protein expression of MCM2-7 were increased in ccRCC compared with normal tissues. High MCM2, MCM4, MCM6 and MCM7 expression were associated with a poor prognosis of ccRCC patients. Functional enrichment analysis revealed that MCM2-7 might influence the progress of ccRCC by regulating the cell cycle. Knockdown of MCM7 can inhibit the proliferation of ccRCC cells. A two-gene risk score including MCM4 and MCM6 can predict overall survival (OS) of ccRCC patients. The risk score was successfully verified by further using Arrayexpress cohort.

**Conclusion:**

We analyze MCM2-7 mRNA and protein levels in ccRCC. MCM7 is determined to promote tumor proliferation. Meanwhile, our study has determined a risk score model composed of MCM2-7 can predict the prognosis of ccRCC patients, which may help future treatment strategies.

## Introduction

Renal cell carcinoma (RCC) is one of the common urinary system tumors (1). It accounts for about 90% of all renal malignancies, of which clear cell renal cell carcinoma (ccRCC) is the most common pathological subtype, accounting for about 75% of renal cell carcinoma (2). The latest statistics show that the incidence of ccRCC is increasing at a rate of 2% per year (3). At present, early ccRCC patients mainly rely on surgical treatment, but most early patients have no specific symptoms, so about 1/3 of patients have already metastasized at the time of diagnosis (4). Patients with metastasis and recurrence not only lose the chance of radical surgery, but also easily tolerate traditional radiotherapy and chemotherapy. Although molecularly targeted drugs have made some progress, most patients will eventually become resistant to targeted drugs. Therefore, mining novel targeted biomarkers related to the diagnosis and treatment of ccRCC is one of the current hot spots in cancer research, and it is also a top priority.

The minichromosome maintenance proteins (MCM) family is a highly conserved DNA unwinding protein complex (5). The MCM protein is an essential replication initiation factor and was originally identified as a protein required for the maintenance of minichromosomes in saccharomyces cerevisiae (6). The most famous of these are MCM2, MCM3, MCM4, MCM5, MCM6, and MCM7, which are composed of six structurally related proteins (7). When MCM2-7 forms a hexamer, it has helicase activity in cells. They are evolutionarily conserved in all eukaryotes. The normal function of MCMs protein is necessary for DNA replication. In the G phase of eukaryotic mitosis, the MCM2-7 complex binds to the chromosome to complete the assembly of the pre-replication complex. After entering the S phase, the MCM2-7 complex is activated by kinase, which induces downstream polymerase-primer synthase assembly. At the same time, it combines with DNA polymerase to form a functional replication fork, which starts DNA unwinding and replication. MCM2-7 must bind to the starting point in the G phase. Without the binding of MCM2-7, DNA replication will not proceed. MCM2-7 is a potential marker of cell proliferation, and an increase in the level of MCM2-7 indicates the proliferation of malignant cells. Accumulating evidence indicates that MCM2-7 is related to the progression and prognosis of malignant tumors (8). However, MCM2-7 remains poorly understood in ccRCC.

This study aimed to provide a systematic and comprehensive study of MCM2-7 gene expression and illuminate their potential roles in ccRCC. We systematically integrated the expression profiles of MCM2-7 and elucidated the potential regulatory mechanisms by bioinformatics analyses. Meanwhile, the personalized prognostic features for ccRCC patients were developed based on MCM2-7 related genes. The research may help to establish a foundation for further intensive MCM2-7- related research in individualized treatment of ccRCC.

## Materials and Methods

### Gene Expression Extraction

RNA sequencing data of ccRCC and clinical data were downloaded from The Cancer Genome Atlas (TCGA) database (https://www.cancer.gov/tcga). According to the previous study, we selected the patients with copy number loss for chromosome 3p, VHL mutation, or both (9, 10). The microarray dataset GSE66272, GSE36895, GSE46699, and GSE53757 were downloaded from the Gene Expression Omnibus (GEO) database (https://www.ncbi.nlm.nih.gov/geo/), which were performed on Affymetrix Human Genome U133 Plus 2.0 Array platform. The method for extracting microarray gene expression values is based on our previous research (11). Then, we used ComBat for batch effect removal to homogenize the gene expression data from GSE66272, GSE36895, GSE46699, and GSE53757. The ccRCC gene expression data of E-MTAB-1980 used for validation cohort were obtained from ArrayExpress (https://www.ebi.ac.uk/arrayexpress).

### Patients and Specimens

The research was composed of 50 ccRCC tissues and 50 normal renal sample from 50 ccRCC patients, who had a renal resection at the Fifth Hospital of Xiamen between June 2019 and January 2021. The standard requirements for patients included in the study were as follows: (1) histologically proven ccRCC; (2) no history of other malignancy tumor; (3) no prior neoadjuvant chemotherapy. The study was performed with the approval of the Ethics Committee of Fifth Hospital of Xiamen and complied with the Helsinki Declaration. Written informed consent was obtained from all patients involved.

### RNA Isolation and RT-qPCR

In the research, total RNA was extracted from ccRCC tissues and cells using TRIzol (Invitrogen, CA, USA) according to the manufacturer’s protocol. Total RNA was reverse transcripted with mRNA reverse transcription kit (Takara, Japan).

RT-qPCR were performed using a SYBR green kit (Vazyme, China) with a Bio-Rad iQ5 (Bio-Rad, USA). Specific primers for RT-qPCR were designed to detect the mRNA level for MCM7 gene. GAPDH was used as internal control to normalize the mRNA levels. All primers were synthesized by Sangon Biotech (Shanghai, China), and the sequences are listed in **Supplementary Table 1**. Relative mRNA level in comparison to controls were determined using 2^−Δct^ method.

### Validation of Protein Expression of the MCM2-7 Genes

The Human Protein Atlas (THPA) provides information on the cell and tissue distribution of 26,000 human proteins. It uses specific antibodies to identify protein expression in tumor tissues and normal tissues. In the research, we explored the protein expression of MCM2-7 genes (MCM2, MCM3, MCM4, MCM5, MCM6, MCM7) in ccRCC tissues and normal tissues.

### Single-Cell Analysis

CancerSEA (12) (http://biocc.hrbmu.edu.cn/CancerSEA/) depicts single cell functional status maps that contain 14 functional states (including stemness, proliferation, EMT, differentiation, invasion, apoptosis, DNA damage, hypoxia, metastasis, angiogenesis, DNA repair, inflammation, quiescence, and cell cycle) obtained from 25 types of tumors including 41900 individual cells. In the research, CancerSEA was used to evaluate the potential roles of MCM2-7 genes in ccRCC.

### Weighted Gene Correlation Network Analysis (WGCNA)

WGCNA is a common algorithm used to build gene co-expression networks and was performed by the WGCNA R package. The WGCNA hypotheses that the co-expression gene network follows the scale less distribution firstly, defines the adjoining function of the gene co-expression correlation matrix and gene network formation, the matrix of similarity was constructed using a power of β=3 (4GEOs), β=9 (TCGA) and a scale-free R^2 =^ 0.95. The adjacency matrix was then translated into a topological overlap matrix (TOM). Furthermore, median linkage hierarchical clustering was analyzed using the TOM-based dissimilarity measure with a minimum size of 20.

### Functional Enrichment Analysis

In order to further analyze the specific biological process methods of the potential targets we obtained, we use Metascape (13) (http://metascape.org) to conduct pathway and process enrichment analysis. Metascape is a web-based portal that combines gene, interactome analysis and annotation functional enrichment.

### LASSO Analysis

The Least absolute shrinkage and selection operator (LASSO) was conducted to construct MCM2-7 genes risk predictive model with the help of “survival” and “glmnet” packages in R software. LASSO is a common method used in high-dimensional data regression, which can select prognosis-related gene pairs of ccRCC by shrinking regression coefficients. The optimal penalty weight of the Lasso-Cox model was found in a grid search manner in a 10-fold cross-validation process. Then, the coefficients of most gene pairs reduced to zero, and a small number of gene pairs with nonzero coefficients were closely correlated with the prognosis of ccRCC.

### DepMap

The Cancer Dependency Map (https://depmap.org/portal/) developed CERES, a computational method to estimate gene-dependency levels from CRISPR-Cas9 essentiality screens while accounting for the copy number-specific effect (14, 15). Dependency scores for MCM2-7 genes in RCC cells were calculated using the CERES algorithm. A negative score indicates that the cell line grows slower when the specific gene is knocked down, while a positive score indicates that the cell line grows faster after the specific gene is knocked down (16).

### Cells Culture and Transfection

The cell lines 786-O and A-498 were obtained from American Type Culture Collection (ATCC, Manassas, VA, USA). 786-O cells and A-498 cells were respectively cultured in PRMI 1640 (Gibco by Life Technologies, Grand Island, NY, USA) and MEM Alpha medium (Gibco by Life Technologies, Grand Island, NY, USA) containing 10% fetal bovine serum (FBS, BI, Kibbutz Beit Haemek, Israel) and 100 U/mL penicillin and 0.1 mg/mL streptomycin (BBI life sciences, shanghai, China) at 37 °C in a humidified incubator with 5% CO_2_. The sequence of shRNA targeting MCM7 was cloned into pLVX vector. The transfection was performed using lipofectamine 2000 (Invitrogen, Carlsbad, CA, USA) according to the manufacturer’ s guidelines.

### CCK-8 Assay

Cell proliferation was measured by CCK-8 assay. 786-O and A-498 cells were seeded onto five 96-well plates (2×10^4^ cells/well) in triplicate and cultured for 24, 48 and 72 hours. Four hours before absorbance measuring, 10 μL CCK-8 solution was added. The absorbance was measured at 450 nm with a microplate reader after incubated at 37°C for 2 hours.

### Drug Sensitivity Evaluation

GSCALite (http://bioinfo.life.hust.edu.cn/web/GSCALite/) is a website used for genomic cancer analysis and drug sensitivity analysis. In the research, we use the GSCALite database to evaluate the drug sensitivity of MCM2-7 genes to identify potential molecular compounds for targeted immunotherapy.

### Statistical Analysis

In this research, a statistical correlation was calculated by t-test. Unpaired samples used unpaired t-test and paired samples used paired t-test. Overall survival (OS) was evaluated by the Kaplan-Meier method, and survival curves were compared by log-rank test. All p values < 0.05 were considered statistically significant.

## Results

### The Expression and the Prognostic Significance of MCM2-7 in ccRCC

**Figure 1:**
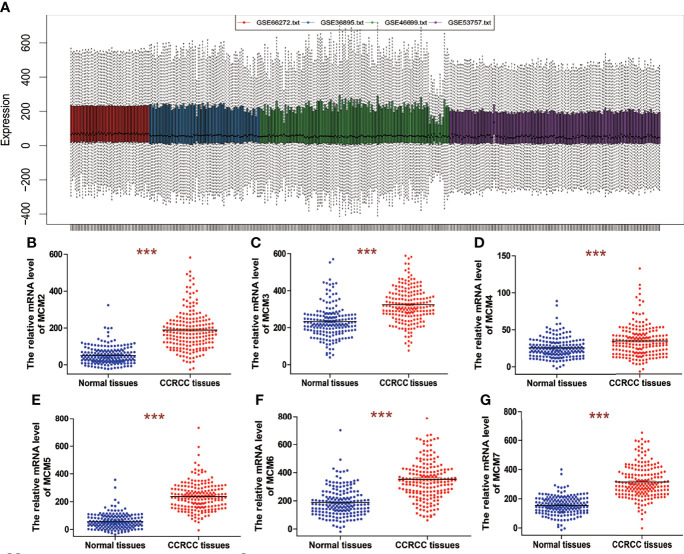
The mRNA expression of MCM2-7 in ccRCC. **(A)** Removing batch effect between GSE66272, GSE36895, GSE46699 and GSE53757. **(B–G)** The expression of MCM2 **(B)**, MCM3 **(C)**, MCM4 **(D)**, MCM5 **(E)**, MCM6 **(F)** and MCM7 **(G)** in ccRCC tissues compared with normal tissues. ***p < 0.001.

Four datasets, including GSE66272, GSE36895, GSE46699, and GSE53757, were obtained from GEO. GSE66272 contains 27 ccRCC tissues and 27 paired adjacent normal kidney tissues. GSE46699 contains 29 ccRCC tissues and 23 adjacent normal kidney tissues. GSE46699 contains 67 ccRCC tissues and 63 adjacent normal kidney tissues. GSE53757 contains 72 ccRCC tissues and 72 paired adjacent normal kidney tissues. The platform used to generate data for the four datasets was the Affymetrix Human Genome U133 Plus 2.0 Array. Before the identifying of MCM2-7 expression, the normalization and batch effects removal from the four GEO datasets was conducted by the ‘sva’ package and was named as 4GEOs (**Figures 1A**).

**Figure 2:**
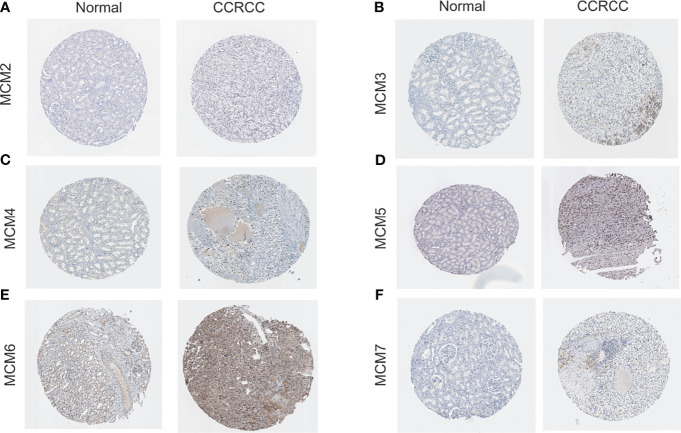
The protein expression of MCM2-7 in ccRCC. **(A–F)** Representative immunohistochemistry analysis of MCM2 **(A)**, MCM3 **(B)**, MCM4 **(C)**, MCM5 **(D)**, MCM6 **(E)** and MCM7 **(F)** from the human protein atlas database.

We analyzed overall expression differences in MCM2-7 genes using 4GEOs. As shown in **Figures 1B–G**, the expression of the MCM2-7 genes in the ccRCC tissues was significantly higher than that in normal kidney tissues. In addition, we examined the IHC results from the Human Protein Atlas project and found that MCM2 was lowly expressed in ccRCC tissues but no detectable expression in normal tissues (**Figures 2A**), MCM3 has moderately expressed in ccRCC tissues but no detectable expression in normal tissues (**Figures 2B**), MCM4 was highly expressed in ccRCC tissues but moderately expressed in normal tissues (**Figures 2C**), MCM5 was moderately expressed in ccRCC tissues but no detectable expression in normal tissues (**Figures 2D**), MCM6 was moderately expressed in ccRCC tissues but lowly expressed in normal tissues (**Figures 2E**), MCM7 was lowly expressed in ccRCC tissues but no detectable expression in normal tissues (**Figures 2F**). These results indicated that the mRNA and protein level of MCM2-7 were increased in ccRCC tissues compared with normal kidney tissues.

**Figure 3:**
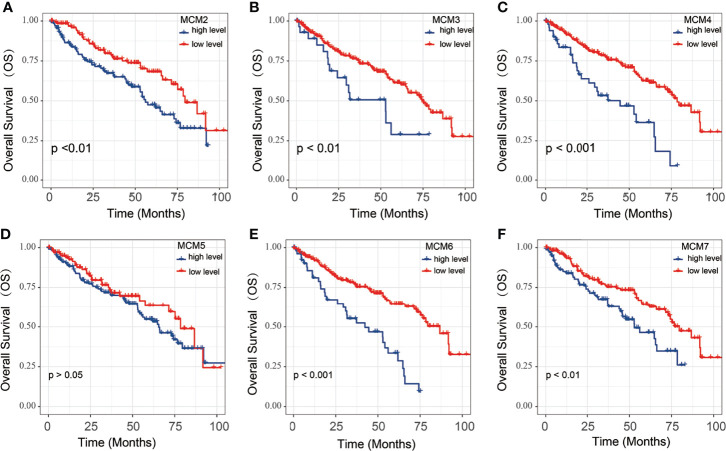
The OS of MCM2-7 for ccRCC patients. **(A–F)** Overall analysis for the prognostic value of MCM2 **(A)**, MCM3 **(B)**, MCM4 **(C)**, MCM5 **(D)**, MCM6 **(E)** and MCM7 **(F)** expression for OS in ccRCC patients by Kaplan-Meier analysis based on TCGA. The Kaplan-Meier method was used to draw survival curves, and the log-rank test was performed to evaluate survival difference with the best cut-off value.

To explore the significance of MCMs gene members in clinical prognosis, we used Kaplan-Meier survival analysis to prepare overall survival (OS) for ccRCC patients (**Figures 3**). The Kaplan-Meier survival curve indicated that high MCM2, MCM3, MCM4, MCM6 and MCM7 expression were associated with a poor prognosis of ccRCC; However, the expression of MCM5 was not significantly associated with OS in ccRCC patients.

### MCM2-7 Were Associated With Cell Cycle Activated Pathways in ccRCC

**Figure 4:**
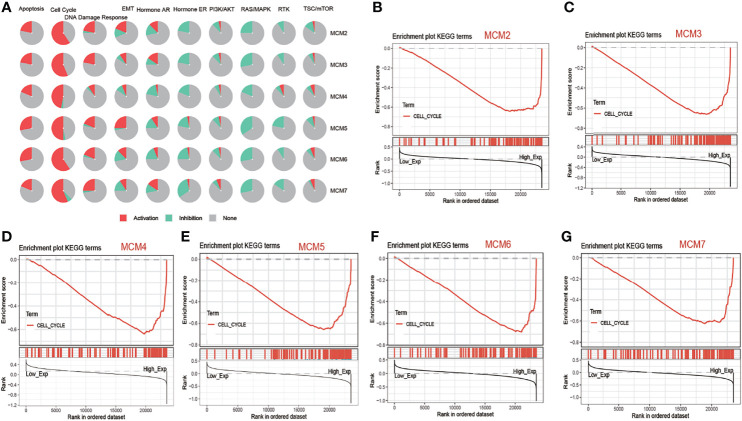
The function of MCM2-7 in ccRCC. **(A)** Pathway activity of MCM2-7 genes from GSCA. **(B)** Enriched gene sets between high and low expression sample groups based on the MCM2 **(B)**, MCM3 **(C)**, MCM4 **(D)**, MCM5 **(E)**, MCM6 **(F)** and MCM7 **(G)** level in ccRCC using GSEA.

Analysis of pathway activity by GSCA revealed that MCM2, MCM3, MCM4, MCM5, MCM6 and MCM7 might strongly activate the apoptosis, cell cycle and DNA damage response as well as inhibit RAS/MAPK pathways and hormone ER (**Figure 4A**). Next, we performed GSEA based on the expression of MCM2-7 to identify the related pathways and underlying mechanisms using 4GEO database. GSEA of TCGA database suggested that MCM2, MCM3, MCM4, MCM5, MCM6 and MCM7 were related to cell cycle (**Figures 4B–G**).

**Figure 5:**
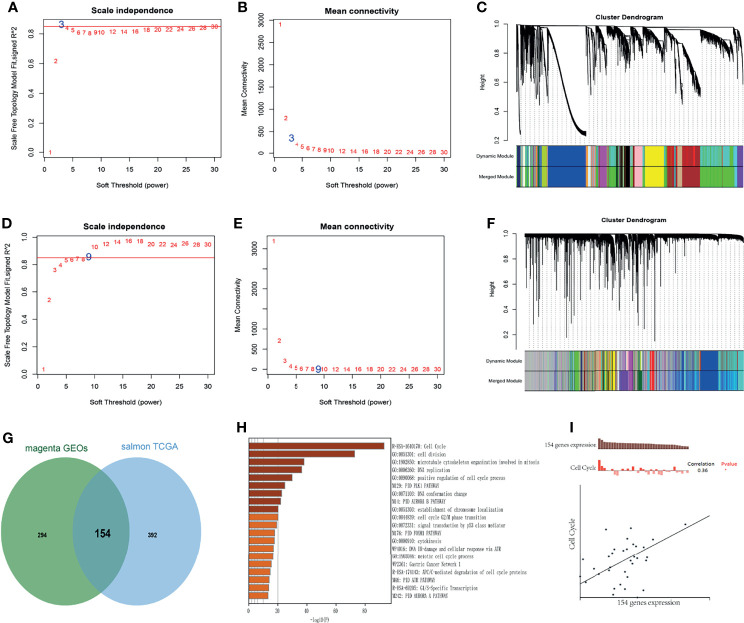
Identification of co-expression module genes associated with MCM2-7 using the WGCNA. **(A)** Relationship between scale-free topology model fit and soft-thresholds (powers) in 4GEOs database. **(B)** Relationship between the mean connectivity and various soft-thresholds in 4GEOs database. **(C)** Dendrogram of modules identified by WGCNA in 4GEOs database. **(D)** Relationship between scale-free topology model fit and soft-thresholds (powers) in TCGA database. **(E)** Relationship between the mean connectivity and various soft-thresholds in TCGA database. **(F)** Dendrogram of modules identified by WGCNA in TCGA database. **(G)** The intersection of magenta module for 4GEOs database and salmon module for TCGA database contained 154 genes. **(H)** Results of KEGG analysis of 154 genes. **(I)** Single-cell analysis indicated that 154 genes were involved in regulation of the cell cycle.

WGCNA was performed to identify the highly correlated genes and co-expression networks of MCM2-7 gene in ccRCC patients. 4GEOs database and TCGA database were used to build WGCNA. We calculated the network topology for soft-thresholding powers from 1 to 30 to choose the best threshold. As shown in **Figures 5A, D**, power value 3 and 9 were the lowest power for the scale-free topology for the 4GEOs and TCGA database respectively. Additionally, soft power showed a higher average connectivity degree (**Figures 5B, E**). Then, we set power=3 (4GEOs) and 9 (TCGA) to produce a hierarchical clustering tree. Modules were identified with a merging threshold of 0.25 (**Figures 5C, F**). Among all modules, the magenta module with 448 genes was the most relevant for MCM2-7 expression in the 4GEOs database, and the salmon module with 546 genes was the most relevant for MCM2-7 expression in the TCGA database. The intersection of the 4GEOs and TCGA databases contained 154 genes (**Figure 5G** and **Supplementary Table 2**). Enrichment analysis revealed that 154 genes were enriched in cell cycle, cell division, microtubule cytoskeleton organization involved in mitosis and DNA replication (**Figure 5H**). The functions of 154 genes in single ccRCC cells were explored *via* CancerSEA and were found to be positively associated with the cell cycle (**Figure 5I**). These results indicated that MCM2-7 would be relevant to ccRCC cells proliferation.

### Knockdown of MCM7 Inhibits ccRCC Cell Proliferation

**Figure 6:**
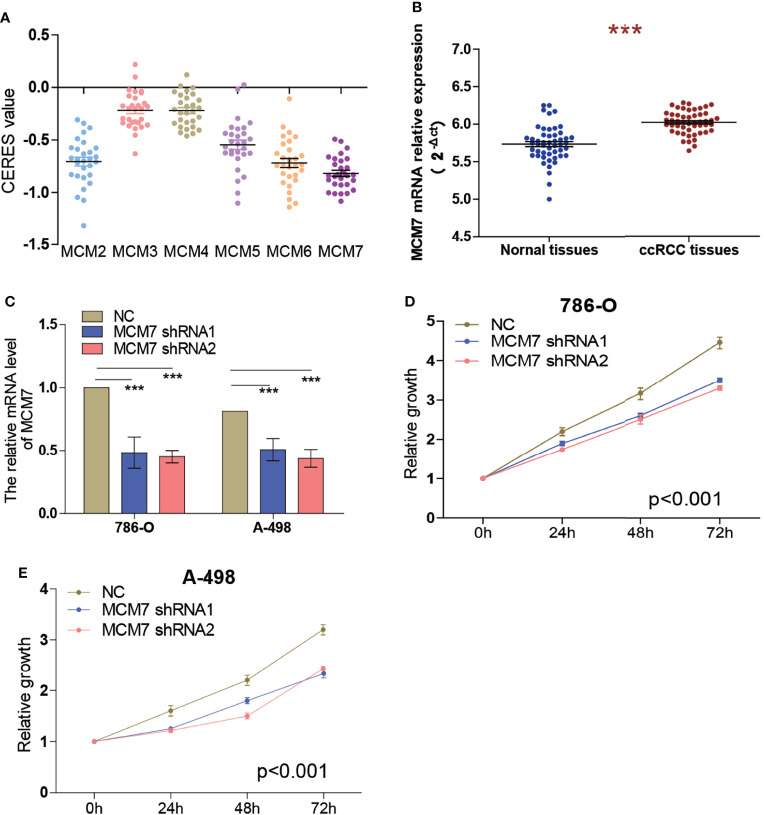
Knockdown of MCM7 inhibits ccRCC cell proliferation. **(A)** Gene effect scores of MCM2-7 in RCC cells from RNAi and CRISPR/Cas9 screens. **(B)** The expression of MCM7 was measured by RT-qPCR from 50 ccRCC patients, results were presented as 2^−ΔCt^. **(C)**The MCM7 expression changes were confirmed by real-time PCR in the ccRCC cells (786-O and A-498) after transfecting shRNAs. **(D, E)** The proliferation ability of 786-O **(D)** and A-498 **(E)** cells was measured by the CCK8 assay after transfecting shRNAs. ***p < 0.001.

To identify the effect of MCM2-7 on the proliferation of ccRCC cells, we investigated genome-wide CRISPR-based loss-of-function screens derived from DepMap. Every gene exhibited different tendencies as potential oncogenes or tumor suppressor genes according to the definition of the CERES, the CERES of MCM2-7 are illustrated in **Figure 6A**. Among six MCMs genes, MCM7 showed a strong tendency towards oncogenes. Next, we examined MCM7 expression in tumor tissues and adjacent normal tissues from 50 ccRCC patients. Results indicated that MCM7 was high expressed in ccRCC tissues compared with normal renal tissues (**Figure 6B**). And then we knocked down MCM7 expression in 786-O and A-498 cells using shRNA (**Figure 6C**). The results of the CCK-8 assay confirmed that MCM7 knockdown significantly inhibited the proliferation of 786-O and A-498 cells (**Figures 6D, E**).

### Prognosis Model of MCM2-7 Genes Constructed and Survival Analysis

**Figure 7:**
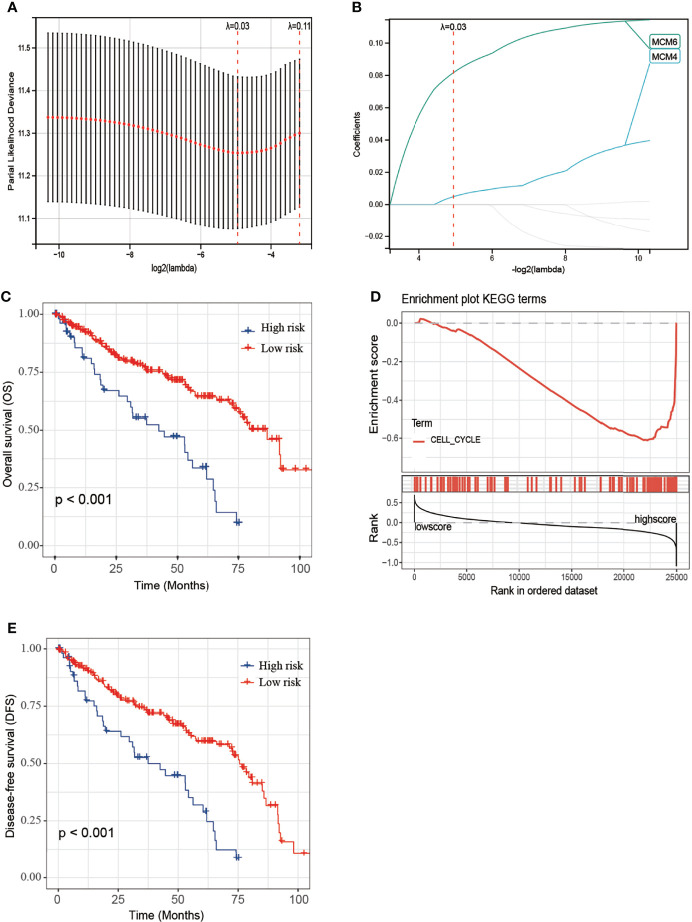
Construction of MCM2-7 genes-based classifier to predict prognosis in ccRCC patients. **(A)** Partial likelihood deviance of OS for the LASSO coefficient profiles. **(B)** LASSO coefficient profiles of MCM2-7 genes for OS. **(C)** Kaplan-Meier curves to compare overall survival of low-risk and high-risk groups. **(E)** Enriched gene sets between high and low score groups in ccRCC using GSEA. **(D)** Kaplan-Meier curves to compare disease-free survival of low-risk and high-risk groups. The Kaplan-Meier method was used to draw survival curves, and the log-rank test was performed to evaluate survival difference with the best cut-off value.

MCM2-7 genes were performed to construct a prognostic model using Lasso-Cox proportional hazards regression (**Figures 7A, B**). The resulting optimal prognostic signature for predicting the overall survival consists of 2 MCM2-7 genes: MCM4 and MCM6. Risk score = (0.005 * MCM4 expression) + (0.081 * MCM6 expression). Each ccRCC patient was assigned a risk score based on the risk score formula and divided into low-risk score and high-risk score groups according to the best cut-off in two groups. Kaplan-Meier curves analysis showed that the overall survival of the low-risk group was substantially longer than the high-risk group (**Figure 7C**). To explore the mechanisms between low score and high score groups, the GSEA approach was performed to identify the potential pathways between different risk score groups. GSEA analysis reveals that the high-risk score group may through involved in cell cycle (**Figure 7D**). Moreover, high-risk score groups also had a worse prognosis in disease-free survival (**Figure 7E**).

**Figure 8:**
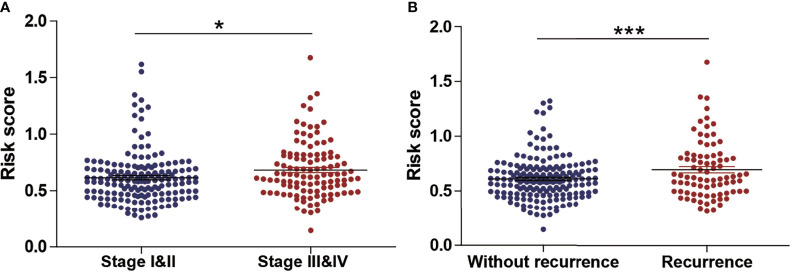
The Relationship Between risk score and Other Clinicopathological Characteristics. **(A, B)** The distribution of risk score in ccRCC patients with low and high stage **(A)**, recurrence and without recurrence **(B)**. NS, p > 0.05; *p < 0.05; ***p < 0.001.

**Figure 9:**
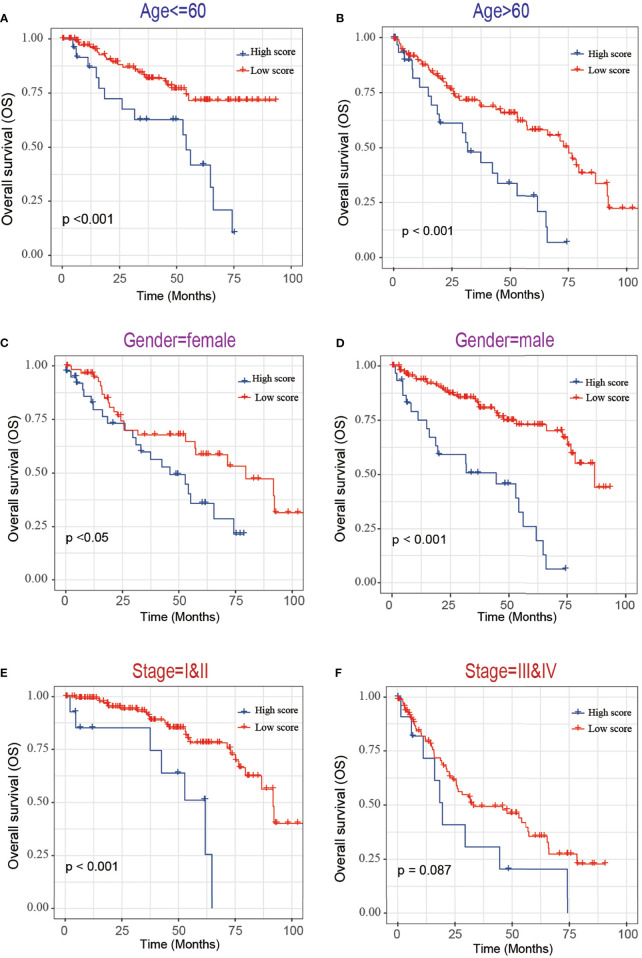
Kaplan-Maier survival curves of overall survival of ccRCC patients according to risk score model in different subgroups. **(A, B)** Prognosis analysis of the ccRCC patients with age<=60 **(A)** and age>60 **(B)** subgroup. **(C, D)** Prognosis analysis of the ccRCC patients with gender=female **(C)** and gender=male **(D)** subgroup. **(E, F)** Prognosis analysis of the ccRCC patients with stage=I&II **(E)** and stage=III& IV **(F)** subgroup. The Kaplan-Meier method was used to draw survival curves, and the log-rank test was performed to evaluate survival differences with the best cut-off value.

Next, we evaluated the association between the risk score and tumor stage or tumor recurrence. **Figure 8A** shows that the risk score was significantly higher in stage III and IV patients than in grade I and II patients. **Figure 8B** shows that the risk score was significantly higher in recurrence patients than in without recurrence patients, indicating that a high-risk score was associated with high malignancy. Moreover, Kaplan-Meier analyses of the patients with low-risk score and high-risk score ccRCC based on clinical factors including age, sex and tumor grade were also performed (**Figure 9**). These results further confirmed the robust stratification ability of the MCM2-7-based risk score.

### Validation of the Prognostic Model

**Figure 10:**
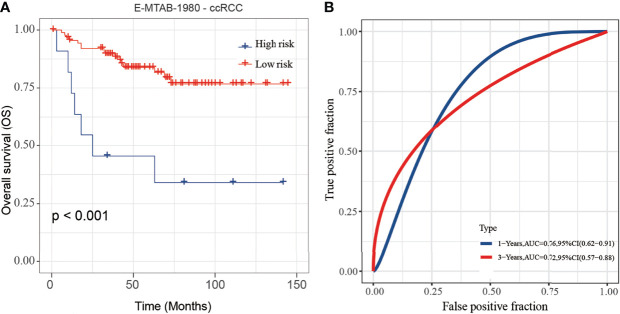
Validation of the risk score model by using E-MTAB-1980-ccRCC database. **(A, B)** The K-M plot **(A)** and 3-year survival ROC curve **(B)** of the risk score model by using E-MTAB-1980-ccRCC database.

To validate the stability and reliability of the prognostic model, we first downloaded 101 samples with complete clinical information as the validation data set from the E-MTAB-1980-ccRCC database. For each patient, the risk score was calculated using the prognostic model. Patients were divided into the low-risk score and high-risk score groups respectively. Kaplan-Meier curve analysis showed that ccRCC patients with high-risk score had a poor OS than those in the low-risk score group (**Figure 10A**). Moreover, the AUC of 3-year OS was 0.71, indicating good accuracy (**Figure 10B**).

### Construction of a Clinical Prognostic Prediction Model

**Figure 11:**
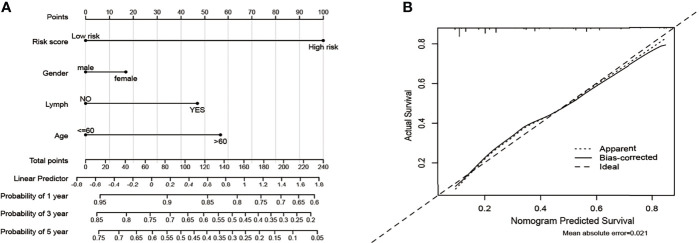
Nomogram and calibration plots for the prediction of outcomes in ccRCC patients based on risk score. **(A)** Nomogram for predicting 1-year, 3-year and 5-year events that combine clinical data with age, gender, lymph, and risk score. The line segment corresponding to each variable is marked with a scale, which represents the value range of the variable, and the length of the line segment reflects the contribution of the factor to the outcome event. The Point in the figure represents the individual score corresponding to each variable under different values, and the total score of the corresponding individual scores after all variables are taken. **(B)** The calibration plots for predicting overall survival.

Based on five variables including age, gender, lymph and risk score, a survival nomogram was created to precisely calculate the probability of survival at 1-year, 3-year and 5-year (**Figure 11A**). The calibration plots suggested that the nomogram performed well in comparison with the ideal model (**Figure 11B**). We hold the opinion that the nomogram may have good accuracy for long-term survival prediction in ccRCC.

### The Relationship of the Drug Sensitivity and MCM2-7

**Figure 12:**
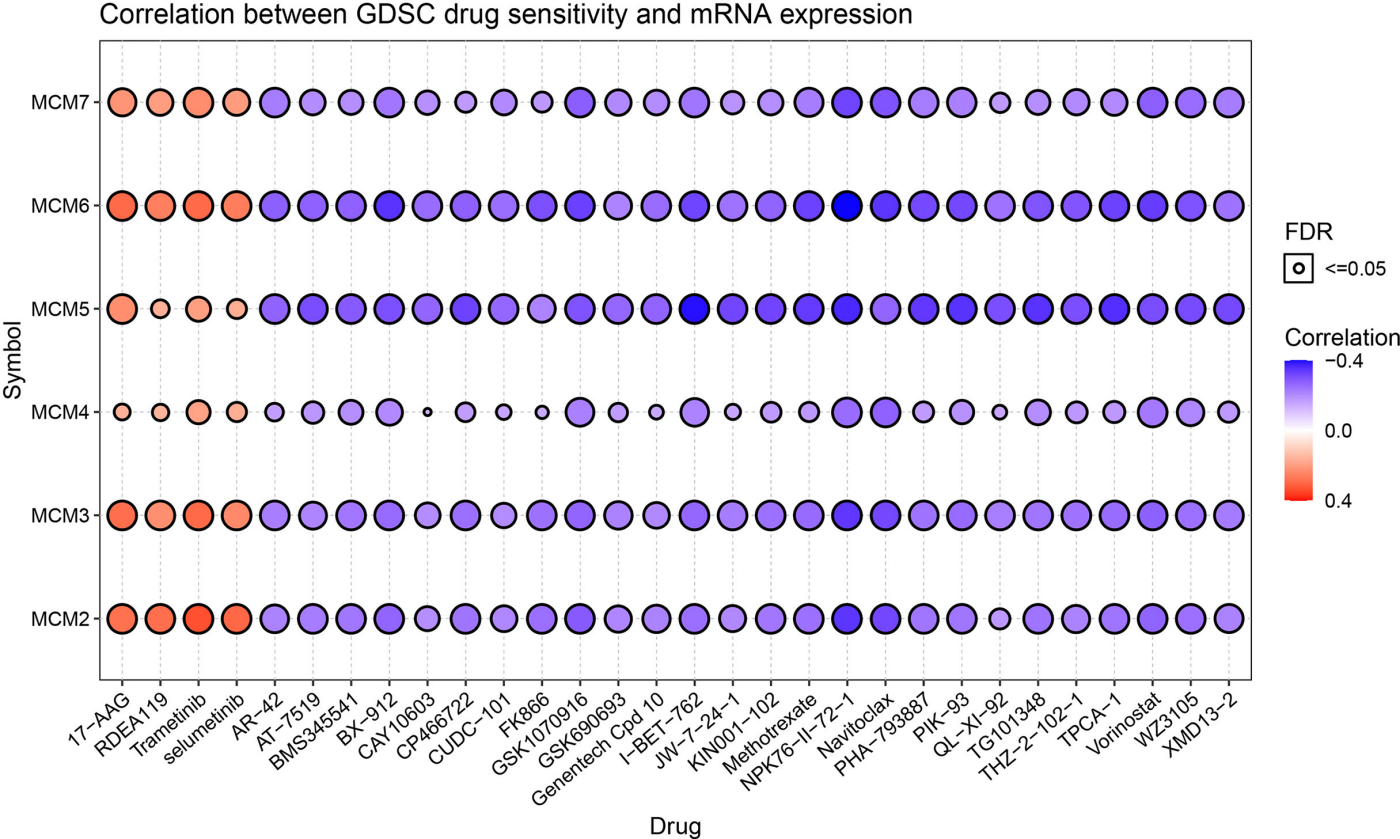
Drug sensitivity of the 6 MCM2-7 genes from GSCA. The correlations between the MCM2-7 expression and drugs. The positive spearman correlation coefficients indicate that high gene expression is resistant to the drug, and vice versa.

The relationship between drug sensitivity and the relative expression levels of MCM2-7 was explored by using GSCA analysis based on the data from the Cancer Drug Sensitivity Genomics Database (GDSC). We hypothesize that a positive correlation between the expression of these genes and the IC50 of the drug being studied will indicate that CCRCC patients develop resistance, and vice versa. High MCM2, MCM3, MCM4, MCM5, MCM6 and MCM7 expression were associated with higher drug resistance to 17-AAG, RDEA119, Trametinib and selumetinib. while there are associated with higher drug sensitivity to AR-42, AT-7519 and BMS345541 et al. (**Figure 12**).

## Discussion

DNA replication is an important issue in the study of tumor occurrence and development. MCM2-7 plays a central role in replication by forming a hexamer ring complex around DNA (17). One of the characteristics of malignant cells is unlimited DNA replication. Therefore, some DNA replication proteins are considered as promising cancer biomarkers. The MCM2-7 is also believed to be closely related to tumor growth and malignant progression. Increased level of MCM2-7 has been detected in various tumor tissues and cell lines.

MCM2 is highly expressed in NSCLC and has certain clinical significance for judging the development and prognosis of NSCLC (18). Knockdown of MCM2 inhibits the expression of cyclin D1 and cyclin-dependent hormone 4 (CDK4), and increases the expression of p21 and p53, indicating that MCM2 silencing triggers cell cycle arrest and induces apoptosis (19). MCM2 is also highly expressed in colorectal cancer and is positively correlated with lymph node metastasis, depth of invasion and Dukes staging (20). In breast cancer, the mRNA and protein levels of MCM2 are increased and tend to increase gradually as the malignancy of tumors increases (21, 22).

Previous studies have shown that overexpression of MCM3 enhances the cell growth, migration, and invasion of medulloblastoma cells (23). Phosphorylated MCM3 promotes the proliferation of renal cancer cells and inhibits apoptosis (24). These results indicate that MCM3 may be related to tumor proliferation, migration, and invasion. Increased level of MCM3 has also been observed in liver cancer, salivary gland tumors, prostate cancer, melanoma, and cervical squamous cell carcinoma (23, 25–28). These studies indicate that MCM3 may be a potential proliferation marker for the diagnosis of certain tumors.

The increased level of MCM4 is related to the development and pathological stages of esophageal cancer (29). The expression of MCM4 is related to the metastasis of melanoma, and related to the poor prognosis of melanoma patients (30). Increased expression of MCM4 is associated with poor prognosis in patients with hepatocellular carcinoma (31). In addition, the down-regulation of MCM4 inhibited the growth, migration and invasion of lung adenocarcinoma cells (32).

The increased level of MCM5 has been found in various cancers, including lung cancer, malignant skin diseases, gastric adenocarcinoma, bladder cancer, oral squamous cell carcinoma, and cervical cancer (33–37). Higher MCM5 level is significantly related to tumor size, histopathological stage, lymph node metastasis and prognosis. In addition, the overexpression of MCM5 can promote the proliferation and invasion of lung cancer cells (38). Knockdown of MCM5 can inhibit the proliferation of renal cell carcinoma (39).

Patients with a high level of MCM6 were found to have poorer survival and a higher risk of death in craniopharyngioma, non-small cell lung cancer, and mantle cell lymphoma (40–42). High MCM6 level is also associated with a higher histological grade in breast cancer, low-grade chondrosarcoma and endometrioid endometrial adenocarcinoma (40, 43).

MCM7 is up-regulated in lung adenocarcinoma and is associated with poor prognosis (44). The up-regulated level of MCM7 is a useful biomarker in the early diagnosis of gastric cancer (45). Knockdown of MCM7 can inhibit the proliferation of gastric cancer cells (46). Inhibition of MCM7 can promote autophagy and apoptosis of skin melanoma cells (47).

Some studies have investigated the expression of MCMs protein in RCC. Expression level of MCM2, MCM5, MCM6, and MCM7 were found to be overexpressed in RCC compared to paired adjacent normal tissue. In addition, a high-level of MCM2, MCM4 or MCM6 was also associated with poorer DFS for RCC patients (48). MCM5 promoted the proliferation of RCC cells and correlates with the progression and prognosis of RCC patients (39). Two studies suggested that MCM2 can be used as a proliferation marker in RCC (49, 50). However, these studies contained all histological types of RCC, including ccRCC, chromophobe RCC, papillary RCC and transitional cell carcinoma. Few studies have been specifically conducted on MCMs expression in ccRCC. Only one study reported MCM6 as a potential prognostic marker in ccRCC (51). To the best of our knowledge, this is the first report investigating the expression levels, co-expression network, potential mechanism of MCM2–7 in ccRCC. This study may have important implications for improving the prognosis of ccRCC patients.

In the study, we analyzed the mRNA and protein expression of MCM2-7 in ccRCC, and the results showed that they were all up-regulated in ccRCC compared with normal tissues. The results of survival analysis demonstrated that a high level of MCM2, MCM3, MCM4, MCM6 and MCM7 were potential factors for the poor prognosis of ccRCC patients.

Next, GSEA analysis was performed to elucidate potential functions of MCM2-7 in ccRCC. Interestingly, for the MCM2-7 high-expression phenotype most gene sets were significantly enriched in the cell cycle pathways. The results indicated that MCM2-7 might influence the progression of ccRCC by regulating the cell cycle. The co-expressed gene of MCM2-7 was identified by WGCNA. The most highly rated gene ontology term of pathway was regulation of cell cycle, which is closely related to cancer growth. The result is plausible because the disturbance in cell cycle can lead to the progression of cancer by affecting chromosomal instability, genomic and proliferation. Other highly ranked terms included cell division, microtubule cytoskeleton organization involved in mitosis, DNA replication and positive regulation of cell cycle process.

Large-scale studies based on CRISPR/Cas9 gene loss-of-function screening provide conditions for studying whether tumor cell proliferation and survival depend on the existence or expression of specific genes. Most tumors have many genetic changes, some of which are considered to be driving factors for tumorigenesis (16, 52). DepMap uses the CRISPR-Cas9 tool to knock out each gene individually to identify candidate genes that are critical to tumor survival. The CERES algorithm was used to calculate the dependency scores of MCM2-7 genes, and the results showed that among the 6 genes, MCM7 was the most essential gene. Therefore, we knocked down MCM7 in ccRCC cells and observed its effect on cell proliferation. The results showed that knocking down MCM7 can inhibit the proliferation of ccRCC cells.

In the past few years, studies on single prognostic biomarkers in ccRCC have not been uncommon. However, a single biomarker lacks sufficient credibility to predict the prognosis of patients (53). Therefore, a predictive model composed of multiple biomarkers is necessary.

Using Lasso regression, we constructed a prognostic risk model based on two MCM genes (MCM4 and MCM6). Using this model, each CCRCC patient is assigned a risk score. Subsequently, the model was validated in the E-MTAB-1980-ccRCC validation set. In the validation group, the survival rate of patients with low and high scores was significantly different. The ROC curve and AUC show that the model performs well. In addition, a highly accurate predictive nomogram was constructed integrating the risk score and conventional clinical prognostic parameters including age, gender, and lymph. It could be used to predict the individual 1-year, 3-year and 5-year OS probability for ccRCC patients according to the risk score and other conventional clinical prognostic parameters.

In the study, we demonstrate the relationship between drug sensitivity and the relative expression levels of MCM2-7 using the GSCA. High MCM2-7 expression was associated with higher drug resistance to 17-AAG, RDEA119, Trametinib and selumetinib. RDEA119, Trametinib and selumetinib are MEK inhibitors, 17-AAG is HSP90 inhibitor. Studies have reported that the inhibition of MEK1 leads to a decrease in the phosphorylation of MCM2 and MCM3 (54). Drug sensitivity information can optimize the design of clinical trials by molecular stratification of the patient population.

## Conclusion

Overall, we revealed that high levels of MCM2, MCM3, MCM4, MCM6 and MCM7 were independent adverse prognostic factors. Knockdown of MCM7 can inhibit the proliferation of ccRCC cells. In addition, we have also established a clinically practical and easy-to-implement risk score model consisting of two MCM genes. This model can be a potential biomarker for predicting the prognosis of ccRCC patients.

## Data Availability Statement

The original contributions presented in the study are included in the article/**Supplementary Material**. Further inquiries can be directed to the corresponding authors.

## Ethics Statement

The study was performed with the approval of the Ethics Committee of Fifth Hospital of Xiamen and complied with the Helsinki Declaration. Written informed consent was obtained from all patients involved.

## Author Contributions

JZ and HZ drafted the manuscript. JZ performed the statistical analyses. QW and YW participated in the design of the study. QW and YW conceived of the study. HZ acquired the datasets. All authors read and approved the final manuscript.

## Funding

This work was supported by the Science and Technology Planning Projects of Xiamen Science & Technology Bureau (No. 3502Z20149025).

## Conflict of Interest

The authors declare that the research was conducted in the absence of any commercial or financial relationships that could be construed as a potential conflict of interest.

## Publisher’s Note

All claims expressed in this article are solely those of the authors and do not necessarily represent those of their affiliated organizations, or those of the publisher, the editors and the reviewers. Any product that may be evaluated in this article, or claim that may be made by its manufacturer, is not guaranteed or endorsed by the publisher.
